# The Effects of Artemisinin on the Cytolytic Activity of Natural Killer (NK) Cells

**DOI:** 10.3390/ijms18071600

**Published:** 2017-07-24

**Authors:** Youn Kyung Houh, Kyung Eun Kim, Sunyoung Park, Dae Young Hur, Seonghan Kim, Daejin Kim, Sa Ik Bang, Yoolhee Yang, Hyun Jeong Park, Daeho Cho

**Affiliations:** 1 Nano-Bio Resources Center, Sookmyung Women’s University, Chungpa-Dong 2-Ka, Yongsan-ku, Seoul 140-742, Korea; bobada6025@gmail.com (Y.K.H.); kyungeun@sookmyung.ac.kr (K.E.K.); psy1101@sookmyung.ac.kr (S.P.); 2 Department of Cosmetic Sciences, Sookmyung Women’s University, Chungpa-Dong 2-Ka, Yongsan-ku, Seoul 04310, Korea; 3 Department of Anatomy, Inje University College of Medicine, Busan 614-735, Korea; anato@korea.com (D.Y.H.); kim3062@gmail.com (S.K.); untung10@empas.com (D.K.); 4 Department of Plastic Surgery, Samsung Medical Center, Sungkyunkwan University School of Medicine, 50 Ilwon-dong, Gangnam-gu, Seoul 06351, Korea; si55.bang@samsung.com (S.I.B.); ps7330@naver.com (Y.Y.); 5 Department of Dermatology, Yeouido St. Mary’s Hospital, The Catholic University of Korea, Seoul 150-713, Korea

**Keywords:** natural killer cells, artemisinin, cytotoxicity, degranulation

## Abstract

Artemisinin, a chemical compound used for the treatment of malaria, has been known to show anti-cancer activity. However, the effect of this chemical on natural killer (NK) cells, which are involved in tumor killing, remains unknown. Here, we demonstrate that artemisinin exerts a potent anti-cancer effect by activating NK cells. NK-92MI cells pre-treated with artemisinin were subjected to a cytotoxicity assay using K562 cells. The results showed that artemisinin significantly enhances the cytolytic activity of NK cells in a dose-dependent manner. Additionally, the artemisinin-enhanced cytotoxic effect of NK-92MI cells on tumor cells was accompanied by the stimulation of granule exocytosis, as evidenced by the detection of CD107a expression in NK cells. Moreover, this enhancement of cytotoxicity by artemisinin was also observed in human primary NK cells from peripheral blood. Our results suggest that artemisinin enhances human NK cell cytotoxicity and degranulation. This is the first evidence that artemisinin exerts antitumor activity by enhancing NK cytotoxicity. Therefore, these results provide a deeper understanding of the action of artemisinin and will contribute to the development and application of this class of compounds in cancer treatment strategies.

## 1. Introduction

Artemisinin is a chemical compound extracted from the plant of sweet wormwood (*Artemisia annua* L.), and is a Chinese traditional medicine that has been used in the treatment of malaria [1,2]. Artemisinin is a sesquiterpene lactone, containing an endoperoxide bridge in its chemical structure. The endoperoxide bridge can react with iron to form cytotoxic free radicals, which are considered to be responsible for the anti-malarial activity of the drug. Red blood cells infected with the malarial parasite (*Plasmodium*) have high levels of intracellular free iron, and are therefore susceptible to artemisinin [3]. Cancer cells also characteristically contain higher concentrations of free iron than do normal cells [4]. Artemisinin and its semisynthetic derivatives selectively cause the apoptosis of various cancer cells such as those of colon, breast, lung, and pancreatic cancers [5,6,7]. At low concentrations, artemisinin induces apoptosis in cancer cells. Artemisinin is therefore a potential candidate for the development of cancer therapeutics. However, the effects of artemisinin on immune cells including natural killer (NK) cells, which have the ability to kill tumors, have not been well studied. Here, the effects of artemisinin on NK cells have been investigated.

The NK cell is a cytotoxic lymphocyte that is part of the innate immune system, and can lyse virus-infected and malignantly transformed cells in the absence of prior stimulation [8]. In humans, NK cells comprise up to 15% of peripheral blood mononuclear cells (PBMCs) in circulating blood, and are also found in the liver and placenta. Typically, the activation of NK cells is tightly regulated by a balance between activating and inhibitory signals generated by receptors that discriminate between self and non-self, by monitoring the expression of MHC class I molecules on the target [9]. In human NK cells, activating receptors such as NKG2D and the natural cytotoxicity receptors NKp30, NKp44, and NKp60 recognize ligands expressed on abnormal cells enhancing NK cell activity [10]. One of the major mechanisms underlying killing strategies in NK cells is lytic granule exocytosis. Following the stimulation of activating receptors downstream signaling, lytic granules in NK cells move to the microtubule-organizing center (MTOC) along with the microtubule, and the MTOC then becomes polarized toward the NK-target cell contact area [11,12]. During granule exocytosis, proteins of the soluble N-ethylmaleimide-sensitive factor activating protein receptor (SNARE) family mediate membrane fusion in the immunological synapse [13]. Finally, the secreted granules composed of perforin and granzymes release toward target cells via the immunological synapse [14]. Perforin generates pores on the target cell membrane, and delivers serine protease granzymes inducing apoptosis via a caspase-dependent or -independent pathway [15]. Therefore, there is a significant benefit in developing strategies to induce the exocytosis of lytic granules of NK cells for targeted cancer therapy [10].

This study investigated the function of artemisinin on the cytotoxic effects of NK cells toward cancer cells. We found that artemisinin significantly enhances NK cell cytotoxic activity through granule exocytosis. This provides the first evidence of a potent anti-cancer effect of artemisinin through upregulation of NK activity.

## 2. Results

### 2.1. Artemisinin Enhances the Cytolytic Activity of NK-92MI Cells

Artemisinin induces apoptosis in different types of cancer cells such as breast, lung, and colon cancers, and leukemia [6]. Although the direct anti-cancer effects of artemisinin on various cancer cells are well known, it is still unknown whether artemisinin activates NK cells, which have spontaneous killing effects against cancer cells. 

**Figure 1:**
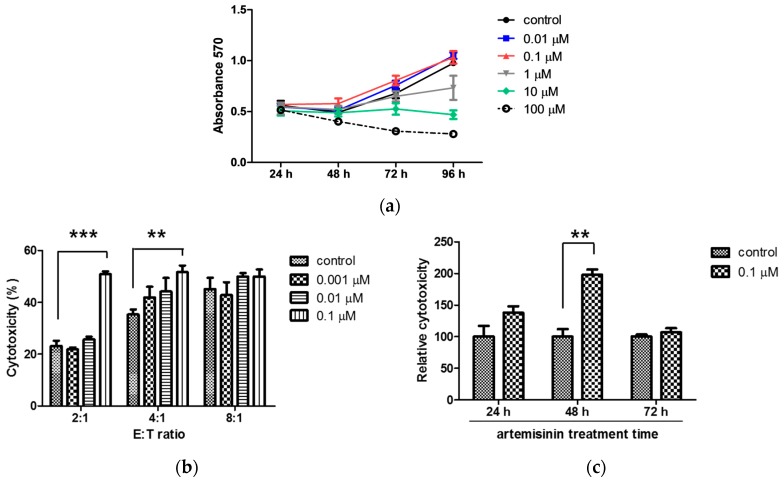
Artemisinin enhances the cytotoxicity of NK cells in a dose-dependent manner. (**a**) NK-92MI cell was treated with 0.01, 0.1, 1, 10, or 100 μM artemisinin, or left untreated, in a 96-well plate. Every 24 h, cell viability was detected using the cell counting kit (CCK)-8 assay up to 96 h. Each line and symbol represents a specific concentration of artemisinin (black circles: 0 μM, blue squares: 0.01 μM, red triangles: 0.1 μM, grey inverted triangles: 10 μM, green diamonds: 10 μM, open circles: 100 μM). (**b**) NK-92MI cells pre-treated with 0.001, 0.01, or 0.1 μM artemisinin for 48 h were co-incubated for 2 h with carboxyfluorescein succinimidyl ester (CFSE)-labeled K562 cells at E/T ratios of 2:1, 4:1, or 8:1. The data shown are representative of three independent experiments (*** *p* < 0.001 versus control, ** *p* < 0.01 versus control). (**c**) NK-92MI cells were treated with 0.1 μM artemisinin for 24, 48, or 72 h and then cytotoxicity assays were performed with K562 cells at E/T ratio of 2:1. The data shown are representative of three independent experiments (** *p* < 0.01 versus control).

To investigate the effects of artemisinin on NK cells, cells from the human NK cell line NK-92MI were treated with various concentrations as indicated in Figure 1a. The result shows that artemisinin had no effect on cell viability at concentrations below 0.1 μM. Therefore, all experiments were carried out at the maximum non-toxic concentration of artemisinin (0.1 μM). To validate the activation of NK cells, NK-92MI cells were treated with 0.001, 0.01, and 0.1 μM of artemisinin for 48 h and then co-incubated with the target cells at various effector/target (E/T) ratios for 2 h. As shown in Figure 1b, at an E/T ratio of 2:1, the cytotoxicity of NK-92MI cells treated with 0.1 μM artemisinin increased by over two-fold in comparison to that of the untreated control group. Treatment with artemisinin significantly enhanced NK-92MI cell cytotoxicity against K562, a human leukemia cell line, in a dose-dependent manner (Figure 1b). The maximum effect of artemisinin on NK-92MI cells was detected after 48 h treatment (Figure 1c). These results suggest that artemisinin directly increases the cytolytic activity of NK cells.

### 2.2. Artemisinin Stimulates Granule Exocytosis of NK Cells

**Figure 2:**
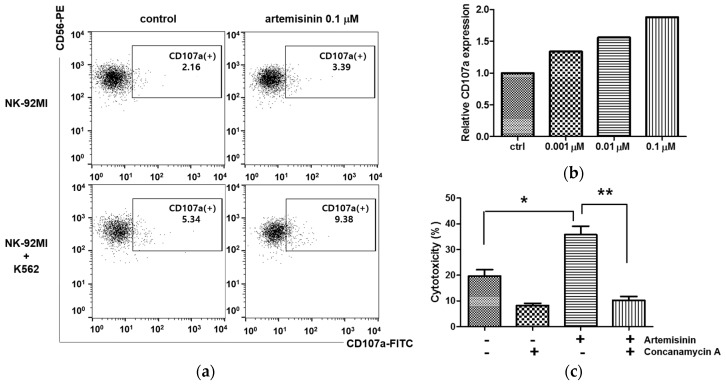
Artemisinin increases cytolytic granule exocytosis in NK cells. NK-92MI cells were treated with 0.001, 0.01, or 0.1 μM artemisinin, or left untreated, for 48 h. The NK cells were then co-incubated with K562 cells at E/T ratio of 2:1 for 3 h and stained with fluorescein isothiocyanate (FITC)-conjugated mouse anti-human CD107a antibody to compare the level of exocytosis. CD107a expression on NK cells was analyzed using BD FACSCalibur. These data are representative of three independent experiments. (**a**) Dot blot shows representative CD107a expression. (**b**) NK-92MI cells pre-treated with 0.001, 0.01, or 0.1 μM artemisinin for 48 h. Bar graph shows the relative CD107a level of artemisinin treated-NK-92MI as compared to the control, set to 1. (**c**) To conduct the inhibitory assay, 0.1 µM artemisinin-stimulated or unstimulated NK cells for 48 h were treated with concanamycin A, or left untreated, for 2 h at 0.01 μM concentration before cytotoxicity. After incubation, NK cells were washed with PBS to eliminate concanamycin A, and then co-incubated with CFSE-labeled K562 cells for the cytotoxicity assay at an E/T ratio of 2:1 (* *p* < 0.05 versus control, ** *p* < 0.01 versus artemisinin 0.1 μM).

It is well known that granule exocytosis is the major mechanism utilized by NK cells for killing tumor cells. Cytolytic granules which contain perforin and granzymes are released during granule exocytosis, presenting lysosomal-associated membrane protein-1 (LAMP-1 or CD107a) on the NK cell membrane [15]. Therefore, detection of CD107a expression on NK cells is regarded as a functional marker for NK cell degranulation and activation [16]. K562 cells were used to stimulate NK cells in the CD107a assay. K562 cells were used to stimulate NK cells in the CD107a assay. As shown in Figure 2a, CD107a expression on the cell surface of K562-stimulated NK cells was increased upon artemisinin treatment, but these levels remained unaffected by artemisinin treatment in the absence of K562 cell stimulation. Relative CD107a expression was increased by artemisinin treatment in a dose-dependent manner (Figure 2b). To confirm that the artemisinin-induced exocytosis effect was associated with enhanced cytotoxic activity, an inhibitory assay was conducted using the degranulation inhibitor concanamycin A, which is a specific inhibitor of V-ATPases [17]. For this assay, cells were treated with 0.01 μM concanamycin A, 2 h before the NK cytotoxicity assay, and then incubated with K562 cells as stimulant. Figure 2c shows that concanamycin A treatment reduced artemisinin-induced NK cytotoxicity to a comparable extent to that of NK cells treated with concanamycin A alone. These data suggest that artemisinin promotes cytolytic activity via the stimulation of granule exocytosis.

### 2.3. Artemisinin Stimulates ERK 1/2 Signaling Down-Stream of Activating Receptor

**Figure 3:**
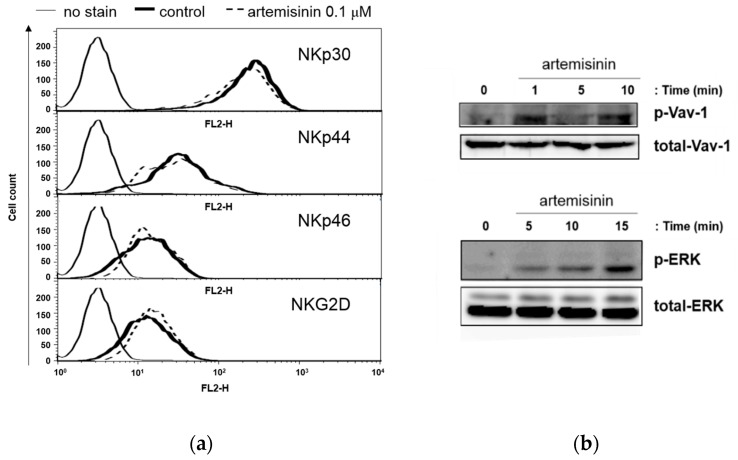
Artemisinin activates activating receptor downstream signal molecules. (**a**) NK-92MI cells treated with 0.1 μM artemisinin for 48 h were stained with antibodies that specifically bind to NKp30, NKp44, NKp46, and NKG2D on the cell surface. The data shown are representative of three independent experiments. (**b**) NK-92MI cells were treated with 0.1 μM artemisinin for 1, 5, or 10 min. Sixty micrograms of each lysate were used to detect total and phosphorylated Vav-1 (upper picture). NK-92MI cells were treated with 0.1 μM artemisinin for 5, 10, or 15 min. Ninety micrograms of each lysate were used to detect total and phosphorylated ERK 1/2 (lower picture). Dimethyl sulfoxide (DMSO) used as a vehicle control does not increase extracellular signal-regulated kinases (ERK) phosphorylation as shown in Figure S1. Details are mentioned at supplementary materials.

To further elucidate the mechanisms underlying artemisinin-enhanced NK cell cytotoxicity, the expression of the NK activating receptors NKp30, NKp44, NKp46, and NKG2D were detected using flow cytometry. NK-92MI cells treated with 0.1 μM artemisinin for 48 h were stained with PE-conjugated-monoclonal antibodies that specifically bind to receptors on NK cells. As shown in Figure 3a, artemisinin did not elevate the expression of these activating receptors on NK-92MI cells. However, even though artemisinin does not change the expression of activating receptors, it may be able to increase the expression of the down-stream signaling molecules. Vav-1, guanine nucleotide exchange factor 1, is a signaling molecule downstream of NK activating receptors such as NKG2D [18]. As shown in Figure 3b, treatment with 0.1 μM artemisinin increased the phosphorylation of Vav-1 within 1 min. In addition, extracellular signal-regulated kinases (ERK) 1/2 phosphorylation was upregulated by artemisinin after 15 min (Figure 3c). ERK is known as a down-stream signal of Vav-1 [19,20]. These results indicate that artemisinin enhances NK cell cytotoxicity not by regulating the expression levels of activating receptors but by inducing activating receptor signals.

### 2.4. Artemisinin Increases Primary Human NK Cell Cytotoxicity

We also examined whether artemisinin increases the cytolytic activity of primary human NK cells. Whole blood samples were obtained from nine healthy donors, and PBLs were isolated using Ficoll density gradient centrifugation. Then, peripheral blood lymphocytes (PBLs) were treated with or without 0.1 μM artemisinin for 48 h. Then, to detect the cytolytic activity of the primary NK cells, NK cytotoxicity assay was performed as described in the Materials and Methods section. Artemisinin did not affect the primary cells’ viability (Figure S2).

**Figure 4:**
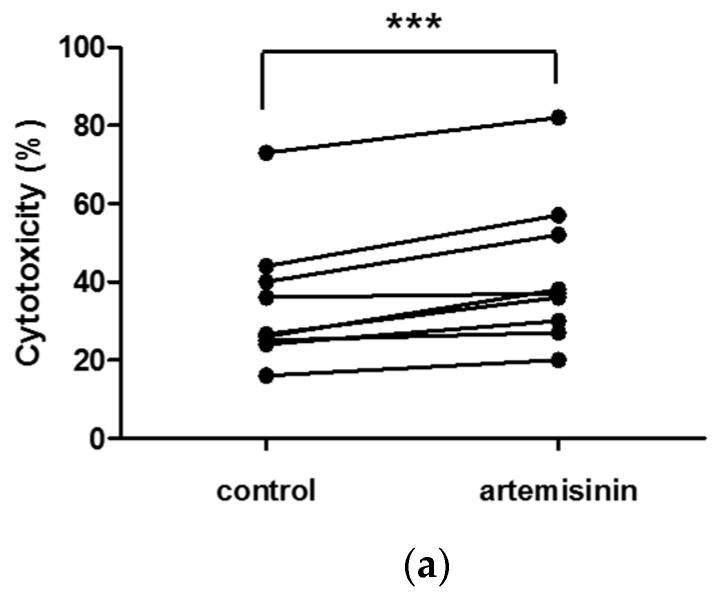

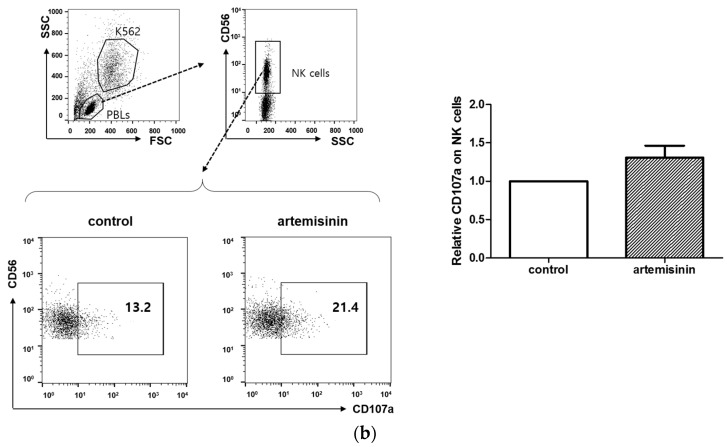
Artemisinin increased cytotoxicity and granule exocytosis in primary human NK cells. (**a**) peripheral blood lymphocytes (PBL)s treated with 0.1 μM artemisinin, or left untreated, for 48 h were co-incubated with CFSE-labeled K562 cells at an E/T ratio of 10:1 for 3 h. Statistical analysis using the paired *t*-test showed that artemisinin stimulates the cytolytic activity of PBLs in most individuals (*** *p* < 0.001). (**b**) PBLs treated with 0.1 μM artemisinin, or left untreated, for 48 h were co-incubated with K562 cells at an E/T ratio of 10:1 with FITC-conjugated anti-CD107a antibody at 37 °C for the CD107a assay. After 3 h of incubation, PE-conjugated anti-CD56 antibody was added for 15 min in order to detect primary NK cells. During analysis, NK cells alone were detected by gating for CD56-positive PBLs and then examining CD107a expression. These data are representative of three independent experiments. The bar graph shows the relative CD107a level in artemisinin-treated PBLs compared to that in untreated control cells, which was set to 1.

Figure 4a shows that artemisinin enhances the cytolytic activity of PBLs. To confirm the degranulation of artemisinin-treated cells, an CD107a assay was performed. As shown in Figure 4b, artemisinin also increased CD107a expression in the NK cells only population, indicating that artemisinin activates cytotoxicity and granule exocytosis in human NK cells.

## 3. Discussion

Artemisinin is an anti-malarial agent extracted from *Artemisia annua*, which has been used in traditional Chinese medicine for a long time [1]. Artemisinin and its synthetic derivatives, such as artesunate and dihydroartemisinin, have also been shown to demonstrate anti-cancer activity in vitro and in vivo [21]. Artemisinin can penetrate through the cellular membrane because of its hydrophobic structure, and exerts anti-cancer effects. Similarly to anti-malarial compounds, the endoperoxide component present in the structure of artemisinin reacts with heme or free irons and generates cytotoxic radicals that induce oxidative damage to cancer cells. The artemisinin family promotes apoptosis via a caspase-dependent pathway in leukemia, or an independent pathway activating BCL2-associated X protein (BAX), a pro-apoptotic molecule, in human colon cancer cells. In addition, artemisinin also exhibits an anti-proliferative effect by blocking cell growth. In prostate cancer, artemisinin inhibits cell division by inducing G1 cell cycle arrest and inhibiting CDK4 gene expression [22]. Similarly, artesunate impedes the growth of breast, lung, and colon cancer, and leukemia [23]. Moreover, artemisinin reduces cancer cell migration and invasion by reducing the expression of matrix metalloprotease (MMP)-2 and MMP-9 and increases the cells’ adhesive ability by enhancing the expression of E-cadherin, resulting in the downregulation of cancer metastasis [24,25]. Artemisinin reduces the levels of factors critical for angiogenesis, such as HIF-1α and VEGF, impeding the supply of cancer environmental factors to cancer cells [26]. 

Although the influence of artemisinin on the immune system remains largely to be investigated, a few immunomodulatory effects of artemisinin have been reported. Neutrophils are a major class of inflammatory cells in the innate immune response, and secrete TNF-α, a pro-inflammatory cytokine. Hunt et al. showed that *Artemisia annua* extract inhibited TNF-α production in activated neutrophils from rats. Inflammation is involved in the development of cancer in terms of inducing a tumor environment. Another important inflammatory cell type, tumor-associated macrophages, also promote tumor progression and metastasis by producing cytokines such as TNF-α, IL-1, IL-6, and transforming growth factor beta (TGF-β) [27]. In addition, dihydroartemisinin inhibits macrophage infiltration and prevents cancer metastasis in epithelial ovarian cancer [28]. However, the effects of artemisinin on innate lymphocytes, specifically NK cells, remains unclear. NK cells are specialized cytotoxic innate lymphocytes that can directly kill both virus-infected and cancer cells. The promotion of NK cell activation and proliferation is used as a critical tool in cancer therapy because of the cell’s natural function of lysing tumor cells without prior activation, unlike cytotoxic T lymphocytes. Therefore, targeting NK cells is an emerging strategy in cancer immunotherapy, and the expansion of highly cytotoxic NK cells constitutes an important clinical approach for cancer therapy [29,30]. 

In this study, we found that artemisinin induced the cytolytic activity of NK cells in a dose-dependent manner as result of enhanced degranulation. It is well known that degranulation is the main mechanism underlying the cytotoxic effect of NK cells. As shown in Figure 3a, the expression levels of representative activating receptors were not affected by artemisinin treatment. However, the phosphorylation of Vav-1, a downstream signaling molecule for NK activating receptors, was quickly and strongly up-regulated. It has been reported that by stimulating activating receptors, Src family kinases stimulate Vav-1, which induces actin polymerase and creates a tight conjugation between NK cells and target cells [31]. Moreover, Vav-1 is known to specifically control ERK activation and exocytosis of cytotoxic granules [32]. In Figure 3b, increased ERK 1/2 phosphorylation was also detected after Vav-1 activation upon artemisinin treatment in NK-92MI cells. These results suggest that artemisinin enhances the degranulation of NK cells via the stimulation of signaling molecules of the NK activating receptor. 

Although we determined that NK cytotoxicity and degranulation was increased by artemisinin at 48 h, the downstream molecules of activating receptors such as Vav-1 and ERK 1/2, was activated within 15 min, implying that some molecules involved in degranulation processes could be modulated at the transcription and/or translational level. Interestingly, expressions of granule content and adhesion receptors were not affected by artemisinin (Figures S3 and S4). Commonly, during lytic granule exocytosis, membrane fusion machinery events are mediated by SNARE family proteins [13]. Studies have investigated the importance of SNARE proteins such as munc18-2, syntaxin 8, and syntaxin 11, on the target killing activity of NK cells and cytotoxic T lymphocytes (CTLs) [33,34,35]. Particularly, NK cells in patients with familial hemophagocytic lymphohistiocytosis type 4 (FHL-4), a disorder caused by mutations in the gene encoding the SNARE protein syntaxin 11 show impaired granule exocytosis and cytotoxicity, implying that syntaxin 11 plays an important role in the degranulation process [36]. Moreover, it has been reported that, in NK cells, IL-2 enhances the expression of syntaxin 11, a SNARE protein that specifically mediates membrane fusion after 72 h [37]. Therefore, it implies that artemisinin might modulate SNARE proteins such as syntaxin 11, resulting in an increase of granule exocytosis and NK cytotoxicity, and further mechanistic studies are required.

An ideal anti-cancer drug should show killing activity specifically on cancer cells with no toxic effects on either normal cells or immune cells. In that respect, artemisinin, a naturally occurring substance from plants, may be an ideal drug, with high effectiveness and low toxicity. Our results have shown that artemisinin may be a powerful anti-cancer drug that acts not only to inhibit cancer progression but also to activate NK cells, which can lyse tumors directly.

## 4. Materials and Methods

### 4.1. Cell Lines and Reagents

A human NK cell line, NK-92MI, was cultured in alpha modified minimum essential medium (MEM alpha; Gibco, BRL, Rockville, MD, USA) supplemented with 2 mM l-glutamine, 0.2 mMi-inositol, 0.02 mM folic acid, 0.1 mM 2-mercaptoethanol (Sigma, St. Louis, MO, USA), 15% fetal bovine serum (FBS; Gibco), and 1% MEM vitamin solution (Gibco). A human leukemia cell line, K562, was used as the target cell line, and was cultured in Roswell Park Memorial Institute medium (RPMI) 1640 (Gibco BRL, Rockville, MD, USA) supplemented with 2 mM l-glutamate and 10% FBS. These cells were maintained in a 5% CO_2_ incubator at 37 °C. Artemisinin (Sigma, St. Louis, MO, USA) was dissolved in dimethyl sulfoxide (DMSO), and added at the respective optimal concentrations for NK-92MI and peripheral blood lymphocytes (PBLs).

### 4.2. Cell Counting Kit (CCK)-8 Assay

NK-92MI cell was treated with 0.01, 0.1, 1, 10, or 100 μM artemisinin, or left untreated, in a 96-well plate. Every 24 h, cell viability was detected using the CCK-8 assay up to 96 h (Dojindo Molecular Technologies, Inc., Rockville, MD, USA). CCK-8 assay was followed manufacturer’s general protocol.

### 4.3. NK Cytotoxicity Assay

The target cells were stained with 1 μM carboxyfluorescein diacetate succinimidylester (CFSE-SE; Molecular Probes Inc., Eugene, OR, USA) for 5 min at 37 °C and then washed three times with cold complete medium. The CFSE-labeled target cells were then incubated at 37 °C with effector cells (NK-92MI or PBLs) that had been pre-treated with artemisinin. After incubation, cells were stained with 7-aminoactinomycin D (7-AAD; BD Pharmingen, San Diego, CA, USA) to detect lysed cells. Cytotoxicity against K562 was analyzed based on regions showing double-positive staining for FL-1 (CFSE) and FL-3 (7-AAD), using a FACSCalibur instrument (Becton Dickison, Franklin Lakes, NJ, USA).

### 4.4. CD107a Degranulation Assay

To quantify NK degranulation, the CD107a degranulation assay was performed using a CD107a detection kit (MBL, Nagoya, Japan). Artemisinin pre-treated NK cells were treated with 6 μg/mL monensin and fluorescein isothiocyanate (FITC)-conjugated mouse anti-human CD107a monoclonal antibody for 3 h at 37 °C. Simultaneously, K562 cells were co-incubated with NK-92MI cells for degranulation stimulation. After incubation, mixed cells were stained with phycoerythrin (PE)-conjugated mouse anti-human CD56 antibody (BD Pharmingen, San Diego, CA, USA) to detect NK cells. CD107a expression on NK cell was analyzed using a FACSCalibur instrument (Becton Dickinson, Franklin Lakes, NJ, USA). 

### 4.5. NKp30, NKp44, NKp46 and NKG2D Staining

To detect the expression of activating receptors, artemisinin-treated NK-92MI cells were stained with PE-conjugated mouse monoclonal antibodies against human NKp30, NKp44, NKp46, or NKG2D (Becton Dickinson, Franklin Lakes, NJ, USA) on ice. After 30 min incubation, the NK-92MI cells were washed twice with PBS, and the expression levels of the receptors were detected using FACSCalibur (Becton Dickinson), with data analysis using FlowJo software (FlowJo LLC, Ashland, OR, USA).

### 4.6. Western Blotting

To confirm whether artemisinin has an effect on the downstream signaling of activating receptors in NK cells, the levels of phosphorylated and total Vav-1 and ERK 1/2 proteins were probed. NK-92MI cells were treated with 0.1 μM artemisinin for 1, 5, 10, or 15 min. After collecting cells, cells were lysed in lysis buffer (Cell Signaling Technology, Danvers, MA, USA) containing PMSF and phosphatase inhibitor cocktail on ice. Proteins were equally loaded on SDS-PAGE, and then transferred onto a PVDF membrane (Bio-Rad, Hercules, CA, USA). Blocking solution containing 5% non-fat dried milk (Santa Cruz Biotechnology, Delaware, CA, USA) was used to block the membrane for 30 min. The membrane was incubated with rabbit polyclonal anti-human antibody to detect total Vav-1 and phosphorylated Vav-1 (Santa Cruz Biotechnology, Delaware, CA, USA), or with rabbit anti-human antibody to detect total ERK 1/2 and phosphorylated ERK 1/2 (Cell Signaling Technology) overnight at 4 °C. After washing with PBS containing 0.1% Tween-20, the membrane was stained with goat anti-rabbit IgG peroxidase (Jackson ImmunoResearch Laboratories, West Grove, PA, USA). Proteins were detected using Amersham ECL Western blotting detection reagent (GE Healthcare, Little Chalfont, UK). The signal was detected using a chemiluminescence-imaging device LAS-3000 (Fujifilm, Tokyo, Japan).

### 4.7. Isolation of Human PBLs

After IRB approval (Inje IRB/1) at Inje University Busan Paik Hospital (Korea), PBLs were obtained from the whole blood of nine healthy donors using Ficoll-Paque Plus (GE Healthcare) density gradient centrifugation methods. Isolated PBLs were maintained in RPMI culture medium (Gibco) supplemented with 2 mM l-glutamate and 10% FBS (Gibco).

## 5. Conclusions

Artemisinin enhances human NK cell cytotoxicity and degranulation. These results provide that artemisinin could be used as cancer treatment strategies by activation of NK cells.

## Supplementary Materials

Supplementary materials can be found at www.mdpi.com/1422-0067/18/7/1600/s1.
