# Supplementary material for: The Effects of Artemisinin on the Cytolytic Activity of Natural Killer (NK) Cells
Source: Int J Mol Sci. 2017 Jul 24;18(7):1600. doi: 10.3390/ijms18071600 (PMC5536087; doi:10.3390/ijms18071600)
Supplement: Supplementary file 1 [file ijms-18-01600-s001.pdf]

Supplementary Materials: The Effects of Artemisinin on Cytolytic Activity of Natural Killer (NK) Cells

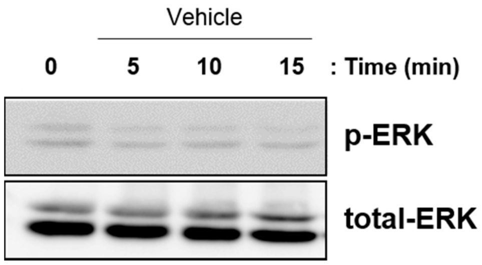

Figure S1. NK-92MI cells were treated with DMSO used as a vehicle control for 5, 10, or 15 minutes were used. Equal amounts of proteins from each sample were loaded. Phosphorylated ERK 1/2 and total ERK 1/2 were detected.

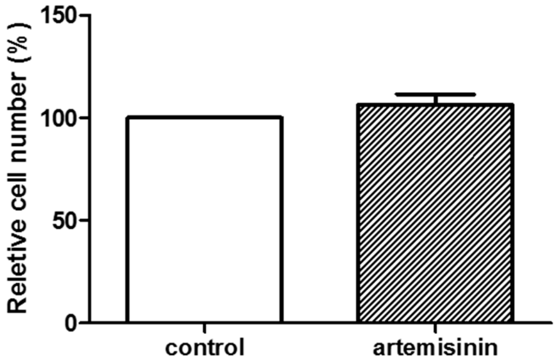

Figure S2. PBLs were isolated from whole blood from nine healthy donors using Ficoll density gradient centrifugation. PBLs were treated with 0.01 μM or 0.1 μM artemisinin, or left untreated, for 48 hours, and then the number of PBLs were counted by the trypan blue staining method. The number of artemisinin-treated PBLs was normalized relative to untreated PBLs.

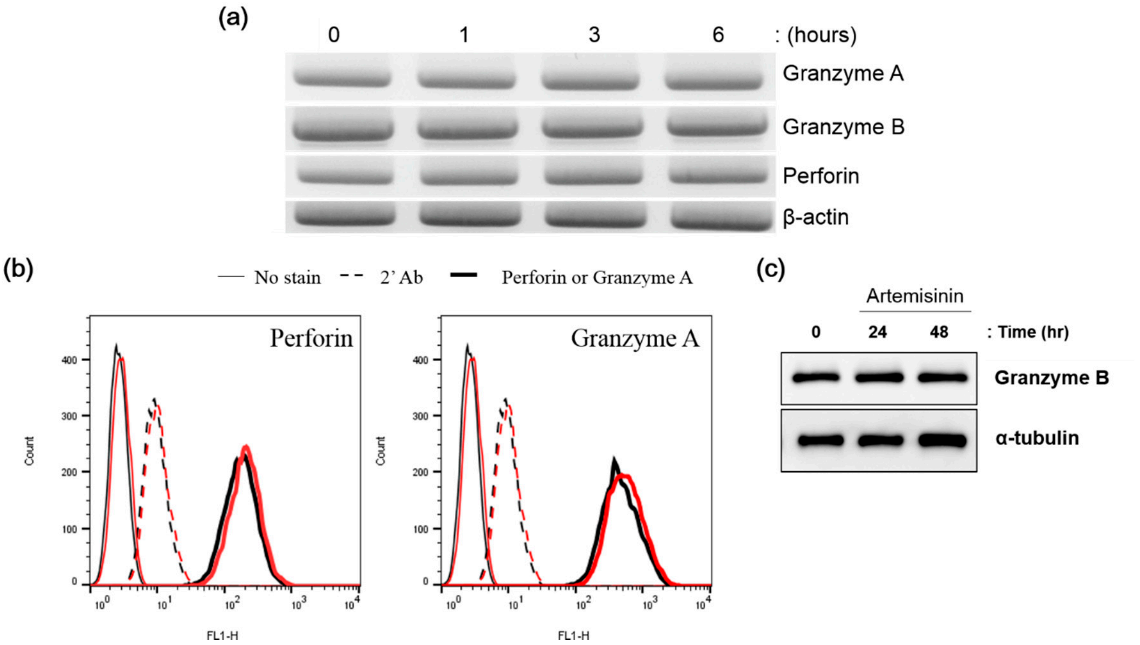

Figure S3. (a) Total RNA was isolated from NK92-MI cells treated with 0.1 μM artemisinin, or left untreated, for 1, 3, or 6 hours, and then cDNA was synthesized. The synthesized cDNAs were used

as templates for PCR, with primers to detect the RNA levels of granzyme A, granzyme B, perforin, and β-actin. (b) NK92-MI cells treated with 0.1 μM artemisinin (red line) or left untreated (black line), for 48 hours were stained with antibodies to detect intracellular protein levels of perforin and granzyme A by flowcytometry. (c) NK92-MI cells were treated with 0.1 μM artemisinin for 24, or 48 hours. Protein levels of granzyme B were detected by western blot assay. The data shown are representative of three independent experiments.

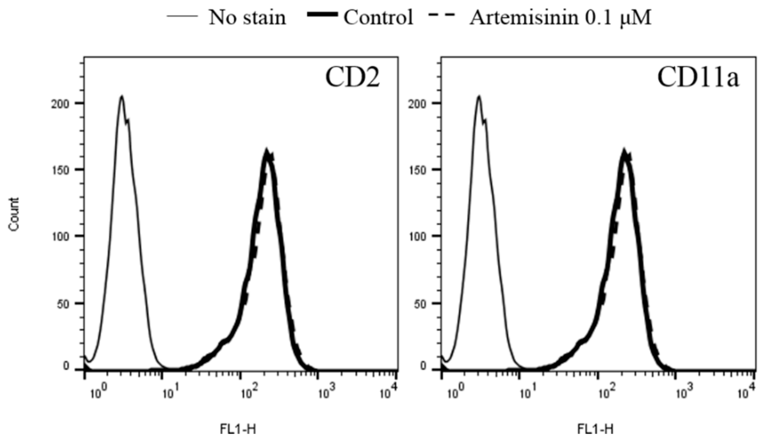

Figure S4. NK92-MI cells treated with 0.1 μM artemisinin for 48 hours were stained with FITC-conjugated-mouse anti-human CD2 or CD11a antibodies for 30 minutes on ice. After washing with PBS, the fluorescence intensity was detected by flow cytometry through an FL-1 filter. The data shown are representative of three independent experiments.

Supplementary Materials and Methods

1. RT-PCR

Total RNA was isolated using the Trizol reagent (Ambion), and then cDNA was synthesized using All-in-One cDNA synthesis SuperMix (Biotool). The synthesized cDNA sequences were used as templates for PCR, with primers to detect RNA levels of perforin (Forward: 5'- GGT GAC CTT CAT CCA AGC AT-3', Reverse: 5'-CTG GCA TGA TAG CGG AAT TT -3'), granzyme A (Forward: 5'-AGC TGA CGG AAA AAG CAA AA -3', Reverse: 5'-TTT CAA GGC CAA AGG AAG TG -3'), granzyme B (Forward: 5'-AAG ACG ACT TCG TGC TGA CA-3', Reverse: 5'-TTC GCA CTT TCG ATC TTC CT -3') and β-actin (Forward: 5'-TCA CCC ACA CTG TGC CCA TCT ACG -3', Reverse: 5'-CAG CGG AAC CGC TCA TTG CCA ATG -3'). PCR reaction was performed with the PCR Master Mix (Bioneer).

2. Flow cytometry

To confirm whether artemisinin influences the expression of adhesion receptors, CD2 and CD11a were detected using FITC-conjugated antibodies (BD Bioscience). NK92-MI cells were treated with 0.1 μM artemisinin, or left untreated, for 48 hours, and then the cells were incubated with mouse anti-human CD2 or CD11a antibodies for 30 minutes on ice. To confirm the levels of proteins present in intracellular granules, cells were fixed and permeabilized with reagents (BD Bioscience), and then incubated with mouse antibodies against human perforin or granzyme A for 30 minutes on ice. FITC-conjugated goat anti-mouse IgG was used as the secondary antibody (BD Bioscience). After washing, the expression levels of adhesion receptors and intracellular granules were detected using FACSCalibur (Becton Dickinson), with data analysis using FlowJo software.

3. Western blotting

Cells were collected and washed with PBS. Cells were lysed in lysis buffer (Cell Signaling Technology) containing PMSF and phosphatase inhibitor cocktail on ice. Equal amounts of proteins

were loaded on 12% SDS-PAGE, and then transferred onto a PVDF membrane (Bio-Rad). Blocking solution containing 5% non-fat dried milk (Santa Cruz Biotechnology, Inc) was used to block the membrane for 30 minutes.

NK-92MI cells were treated with DMSO used as a vehicle control for 5, 10, or 15 minutes were loaded and rabbit anti-human antibodies were used to detect total ERK 1/2 and phosphorylated ERK 1/2 (Cell Signaling Technology) After washing with PBS containing 0.1% Tween-20, the membrane was stained with goat anti-rabbit IgG peroxidase (Jackson ImmunoResearch Laboratories).

To confirm the expression of granzyme B, NK-92MI cells were treated with 100 μM artemisinin for 24, or 48 hours. Ten micrograms of protein from each sample were loaded, and a polyclonal rabbit anti-human granzyme B antibody (Cell Signaling Technology) was used to detect granzyme B levels.

All the proteins used for western blotting were detected using Amersham ECL Western blotting detection reagent (GE Healthcare) and signals were detected using a chemiluminescence-imaging device LAS-3000 (Fujifilm).
